# Land use classification of open-pit mine based on multi-scale segmentation and random forest model

**DOI:** 10.1371/journal.pone.0263870

**Published:** 2022-02-14

**Authors:** Xianyu Yu, Kaixiang Zhang, Yanghui Zhang

**Affiliations:** 1 School of Civil Engineering, Architecture and Environment, Hubei University of Technology, Wuhan, P.R. China; 2 China Railway Siyuan Survey and Design Group CO., LTD, Wuhan, P.R. China; 3 Patent Examination Cooperation Hubei Center of the Patent Office, CNIPA, Wuhan, P.R. China; Universidad de Guadalajara, MEXICO

## Abstract

The mining industry production is an important pillar industry in China, while its extensive production activities have led to several ecological and environmental problems. Earth observation technology using high-resolution satellite imagery can help us efficiently obtain information on surface elements, surveying and monitoring various land occupation issues arising from open-pit mining production activities. Conventional pixel-based interpretation methods for high-resolution remote sensing images are restricted by “salt and pepper” noise caused by environmental factors, making it difficult to meet increasing requirements for monitoring accuracy. With the Jingxiang phosphorus mining area in Jingmen Hubei Province as the studied area, this paper uses a multi-scale segmentation algorithm to extract large-scale main characteristic information using a layered mask method based on the hierarchical structure of the image object. The remaining characteristic elements were classified and extracted in combination with the random forest model and characteristic factors to obtain land occupation information related mining industry production, which was compared with the results of the Classification and Regression Tree model. 23 characteristic factors in three aspects were selected, including spectral, geometric and texture characteristics. The methods employed in this study achieved 86% and 0.78 respectively in overall extraction accuracy analysis and the Kappa coefficient analysis, compared to 79% and 0.68 using the conventional method.

## Introduction

With the continuous development of the Chinese economy, mining production has become an essential part of China’s economic development. However, continuous developments and improvements in the mining economy have also led to several challenges for its sustainable development [1, 2]. Data shows that as of 2015, more than 1.06 million hectares of forested land have been cleared for the exploitation of mineral resources, while 263,000 hectares of grassland have been destroyed. In addition, more than 1.4 million hectares of accessible land resources in China have been directly occupied or indirectly destroyed due to factors such as mining, stacking of tailings, and waste rock. This figure increases by 20,000 hectares each year, while the recovery rate for areas inflicted by geological environmental problems in mining regions is less than 12%.

It is not only that conventional methods for mines monitoring are time-consuming and labor-intensive, in addition, their timeliness and efficiency often cannot be guaranteed. Among many monitoring methods, remote sensing monitoring techniques can be used to rapidly acquire relevant data on the mining development. As this allows for the timely acquisition of accurate data, such techniques are conducive to the surveying and monitoring of various land occupation problems arising from open-pit mining production activities.

The number of high-resolution remote sensing satellites has grown since the turn of the 21st century. At present, the use of high-resolution remote sensing images in remote sensing techniques has become more common in surveying and monitoring for primary mining geological environment problems of various kinds. Conventional image interpretation techniques in remote sensing are mainly “pixel-based”. In other words, such techniques use spectral information stored in pixels to identify features through supervised image classification and unsupervised image classification. These basic image classification techniques for remote sensing have been long been applied in remote sensing techniques for mining surveying and monitoring. For example, Yang used Landsat TM images taken over different periods to compare the efficacy of using supervised image classification and unsupervised image classification to observe changes in land use in mining regions. The final results show that supervised image classification and unsupervised image classification lead to different classifications for different specific features. Classification methods should be adapted as appropriate for different situations [3]. After entering the era of high-resolution remote sensing images, many researchers have continued to study the application of supervised image classification and unsupervised image classification methods in remote sensing techniques for mining surveying and monitoring. For example, scholars such as Mezned have integrated the SPOT-5 panchromatic band and Landsat ETM+ multi-spectral data based on a remote sensing technique involving multi-spectral and multi-source data fusion to enhance the different display of tailing piles and tailings ponds in mining regions, thus obtaining the linear spectrum of mixed images. Then the results obtained through unsupervised classification can guarantee the authenticity, indicating that this method can be applied to long-term multi-temporal monitoring of tailing slags and tailing ponds [4]. In 2017, Chen used Chinese GF-1 data to compare the performance of several supervised classification methods applied to land use and land cover, including the maximum likelihood method, minimum distance method, and support vector machines, so as to discuss the differences in such supervised classification methods in applications of high-resolution images [5].

More researchers began to apply high-resolution remote sensing satellite images such as GF-1 and GF-2 of China to Mining remote sensing survey and monitoring research work. By analysing the spectral and spatial characteristics of surface features, using band calculations or density segmentation algorithms, they comprehensively analysed the land use status of mining areas, and investigated and analysed the development status of mineral resources and environmental changes; besides, they also established a set of mine remote sensing monitoring interpretation signs based on high-resolution remote sensing images [6–10].

However, the extraction of information on feature elements from high-resolution images using conventional remote sensing interpretation methods can only provide pixel-level spectral characteristics. The factors that represent characteristics and internal associations of objects such as textures and shapes cannot be fully utilized. Meanwhile, conventional methods cannot avoid “salt and pepper” noise caused by massive image information or the degradation of classifier performance caused by information redundancy [5]. To solve this problem, researchers in various countries have studied techniques for extracting information from high-resolution remote sensing images with respect to image object construction and object classification. The former is based mainly on the proper segmentation of remote sensing images, while the latter is obtained through research accomplishments related to relevant classification algorithms, such as supervised classification and unsupervised classification [11, 12].

Developments in computing capabilities have led to constant updating of various segmentation algorithms. In particular, multi-scale segmentation algorithms are unparalleled in both range and performance. Segl and Kaufmann combined supervised shape classification and unsupervised image segmentation in an iterative procedure, deriving a method that can detect small objects in high spatial resolution panchromatic images and allowing target-oriented search for specific object shapes [13]. Chen and Lee introduced the expectation maximization algorithm to modify multi-resolution wavelet analysis model, proposing a segmentation algorithm for image classification that effectively achieves the texture segmentation of experimental images [14]. To quantitatively determine the optimal segmentation scale, Wang, Dong, and Chen proposed an image segmentation method combining superpixels and minimum spanning trees to seek the best image segmentation results. They compared their extraction method with the two other segmentation algorithms, which allowed them to obtain the best segmentation effect [15]. Li, Tang, and Liu proposed a multi-scale image segmentation method in remote sensing based on an improved minimum spanning tree. Their method considered the regional merging of shape parameters and multi-band spectral characteristics. The principle of the minimum heterogeneity is core to their method. In addition, their tests show that this method is superior to multi-scale segmentation methods integrated using the eCognition software in terms of subdivision in areas with small spectral differences [16]. However, the main consideration in such studies lies in how to optimize the segmentation of image object units, with only a few representative factors selectable from the spectral, texture and geometric information to extract specific features, which cannot achieve the separation of multiple elements.

Therefore, some scholars have begun to use machine learning algorithms to solve the above problems in multi-scale segmentation researches. In particular, tree machine learning algorithms, represented by decision trees, classification regression trees, random forests and rotation forests, have been widely applied in research on remote sensing for image classifications, as they have clear model structures and simple model parameters. For example, in a study based on tree ensemble learning algorithms, Acharya, Lee and Yang used the open-source decision tree J48 to explore the effect of using Landsant8 OLI images to identify water bodies. In addition to conducting a statistical analysis of their model with the Kappa statistic and the area under the curve (AUC) methods, they also compared their results with five other water body identification methods. Their results show that their decision tree model can be applied to the research of identifying water bodies with different resolutions in a broader range [17]. Pelletier, Valero and Inglada compared the application of the random forest model and the support vector machine model in land cover classifications in southern France, using SPOT4 and Landsat8 images as data sources. Their final experimental results show that the overall accuracy of the random forest model is 83.3%, greater than that of the support vector machine model, while the random forest model is superior to the support vector machine model in terms of parameterization and training time [18]. Through such studies, issues related to the extraction of multi-target information can be resolved to some extent, and noise generated from the classification of pixel objects can be avoided. However, extraction is less than ideal for small target elements when there are great differences in the area of feature elements.

The advantage of tree models is that they are easy to understand and relatively difficult to overfit. Although these models can accommodate small datasets for several landslide susceptibility mapping cases, when the number of samples or the factor dimension increases, the accuracy of the results is substantially influenced [19–22]. Dou et al. simulated landslide occurrences induced by massive rainfall on Izu-oshima Volcanic Island in Japan using decision tree and random forest models and reported that the random forest models had good robustness [23]. Pourghasemi and Rahmati compared the results of 10 machine learning models for landslide susceptibility mapping in the Ghaemshahr Region, Iran, and the random forest model was found to have the best performance [21].

The main approach taken in this paper is to extract characteristic rule sets from the spectral, texture and geometric information for different feature elements, and then gradually separate and extract various feature elements. Therefore, in this paper, we introduce an image object inheritance hierarchy to identify the image segmentation objects. The optimal segmentation scale is calculated and evaluated for different objects using the Euclidean Distance 2 Index. We establish a set of methods for extracting the land occupation elements of open-pit mining production from the high-resolution images in remote sensing by combining a multi-scale segmentation algorithm with a tree model classifier, making the extracted image information more scientific, accurate and reliable.

## Overview of studied area and data sources

### Overview of test area

**Fig 1:**
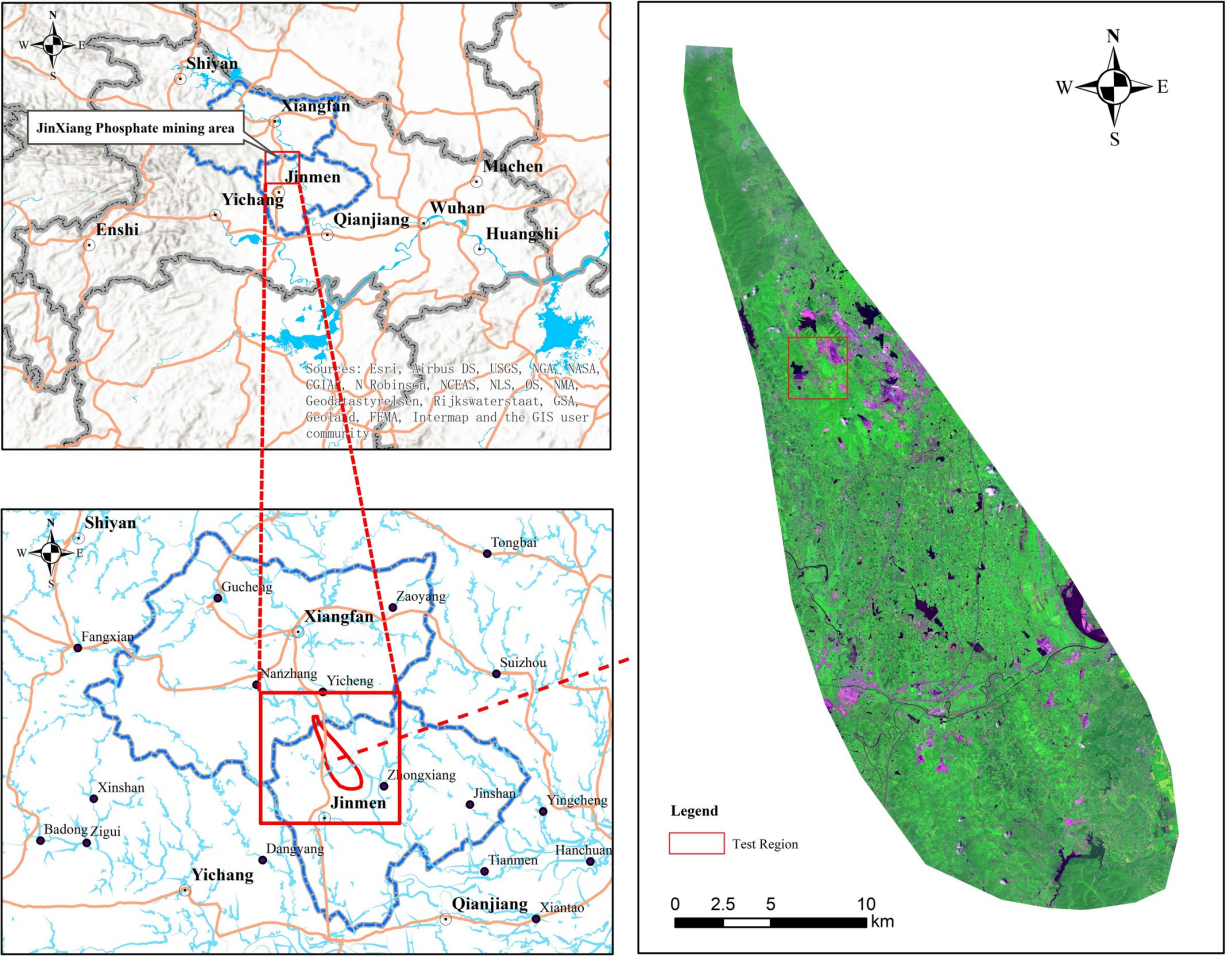
Schematic diagram of the research area of Jingxiang phosphate mine. Republished under a CC BY license, with permission from HBUT, original copyright 2017.

Our paper focuses on the Jingxiang phosphorus mining area in Jingmen, Hubei (as shown in Fig 1). In terms of topography, the mining areas located on Yicheng (Xiangyang) and Zhongxiang (Jingmen), Hubei Province; the concentrated mining area of the Jingxiang phosphorus mining area is located in the middle reaches of the Han River, the southern foot of Dahong Mountain, the transition zone from the mountainous areas of northern Hubei to the Jianghan Plain. The terrain in this area slopes down from west to east, with altitudes varying between 40 m to 418 m. In terms of fundamental geological characteristics, the lithology of the studied area is dominated by the overlying Sinian from the Proterozoic-Archaean crystalline base, or the Mesoproterozoic strata, the Paleozoic sedimentary rock strata and the Permian strata. There are 6 proven categories covering 27 kinds, including phosphate rock, rectorite, pyrite, aluminum, silicon, and barite. In particular, this area has 536 million tons of phosphate rock reserves, the second-largest of exploitable reserves in China [24].

In this paper, a block located in the northern part of the studied area is selected as the test area, while the rest of the studied area is classified as the training area. The data from training area is used to train the classifier model, before using data from the test area for model performance tests.

### Data sources

The research of this paper is mainly based on full-band image data taken by GF-2 satellites in the studied area in 2017 and supplemented by data taken by the 30 m Advanced Spaceborne Thermal Emission and Reflection Radiometer Global Digital Elevation Model (ASTER_GDEM) in Hubei, as well as the information taken from the 2017 mining rights in the studied area, as shown in Table 1. Among them, the GF-2 satellite is a civilian remote sensing satellite with the highest spatial resolution launched by China on August 19, 2014. The Satellite has a panchromatic resolution of 0.8 meters and a multi-spectral resolution of 4 meters, featuring high resolution, low orbital height, short regression period, high image accuracy, and strong side swing mobility.

**Table 1:** Data resources.

Data Source	Data Time	Resolution	Type
**GF-2**	2017	0.8m(Panchromatic), 4m(Multispectral)	Raster
**ASTER_GDEM**	/	30m	Raster
**Mineral Rights data of Hubei Province**	2016–2017	/	Vector
**OSM Geographic information data**	2017	/	Vector

## Research methods

**Fig 2:**
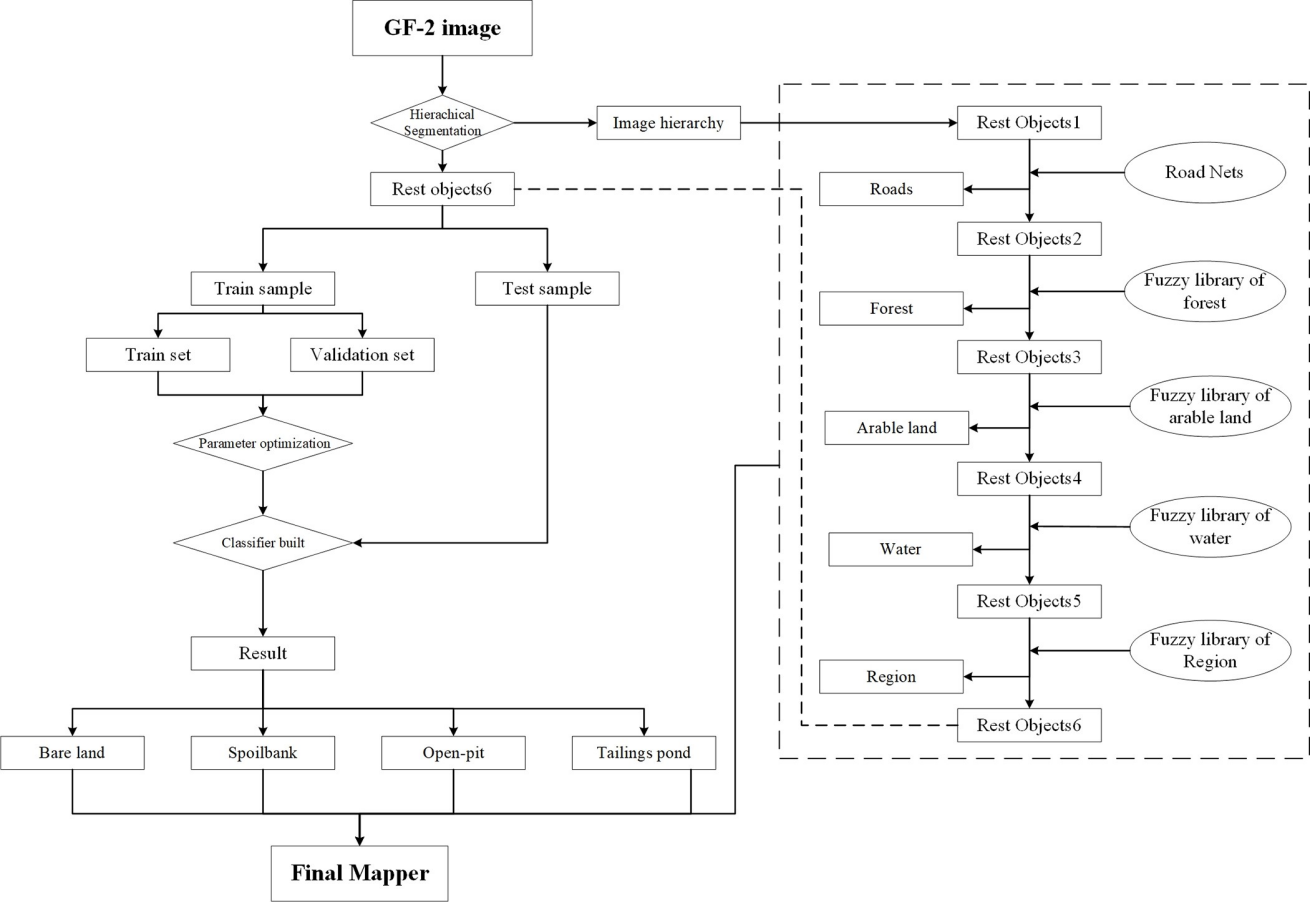
Method flow chart of this experiment.

To solve the problem of extracting land occupation information related to open-pit mining development from high-resolution images acquired through remote sensing techniques, it is necessary to obtain the optimal segmentation scales at different hierarchies through multi-scale segmentation and the collection of data [25, 26]. Thereafter, the information extraction rules for each layer should be determined through the calculation and assessment of characteristic factors, image object hierarchy to establish an overall information extraction rule set [27–29]. After a wide range of geological background elements are removed, the geological environment background problem factors and the residual geological environment background factors regarding various open-pit mining developments contained in the remaining remote sensing images and that are to be extracted should be extracted using the machine learning algorithms, as shown in Fig 2.

In order to analyse and compare single classifier and ensemble learning classifiers, the machine learning algorithms used in this experiment include Classification and Regression Tree model (CART), and Random Forest model (RF), which was set CART as base classifier [30–33]. Meanwhile, this experiment selected confusion matrix, overall classification accuracy and Kappa coefficient as evaluation indicators of the classification results to verify the conclusions of this experiment [34, 35].

## Experimental process

### Selection of ED2-based optimal segmentation parameters

Because of the large test area and the complex and diverse types of features, the Euclidean distance 2 Index (ED2) was used to determine the optimal scale and the decomposition hierarchy for various features. Background elements such as forest land, cultivated land, residential land, water and roads to be used as mining development land were masked to reduce their interference with the extraction of information related to land earmarked for mining developments.

**Fig 3:**
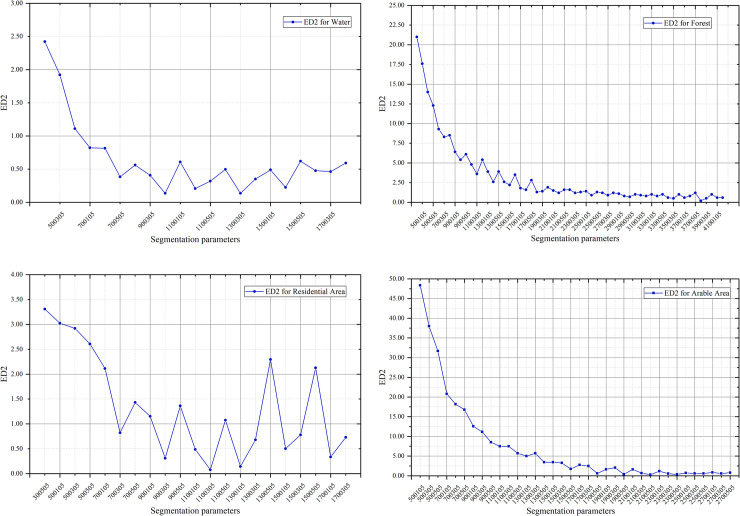
ED2 curves for 4 types of features. (a) ED2 curve of water. (b) ED2 curve of woodland. (c) ED2 curve of residential. (d) ED2 curve of arable land.

First, the road network data was used to mask the main roads, and then samples of various feature types in the ArcGIS software were randomly selected as reference sample polygons and the number and area of the reference polygons were summarized as the parameters for calculating the ED2 index. The weights of the 4 bands of the GF-2 image were set as 1. The division scale range was set to be [50, 270] for arable land, [50, 410] for forest land, and [50, 170] for waters and residential land. The compactness parameter was 0.5, the shape parameter was smaller than 0.5 (i.e. spectral factor ≥ 0.5). The shape parameters were set to be 0.1, 0.3, and 0.5 for the multi-scale segmentation experiment. After the vector map of the segmentation result was loaded into ArcGIS, overlay analysis and calculations were performed for each reference polygon and result vector maps. Finally, the ED2 curves for these 4 types of features, as shown in Fig 3A–3D.

After the shapes and trends of these four curves are compared, the following patterns can be found: when the segmentation scale is very small, the ED2 index is relatively high; when shape and compactness parameters are changed while segmentation scale remains constant, the ED2 index changes accordingly; when the scale increases, ED2 slowly decreases and gradually tends towards stability; when ED2 is close to 0, the shape of the sample and the corresponding segmentation object are closer to each other. The optimal segmentation scale parameters of the 4 types of features, as derived from relevant statistics, are shown in Table 2.

**Table 2:** Optimal segmentation parameters of background elements for each development area.

	Arable Land	Forest	Region	Water
**ED2**	0.19	0.10	0.06	0.13
**Scale**	190	390	110	130
**Shape**	0.5	0.3	0.5	0.3
**Compactness**	0.5	0.5	0.5	0.5

### Establishment of information extraction hierarchy

As shown in Table 3, combining the optimal segmentation parameters and image features of the above-mentioned land categories, the information extraction hierarchies are established in accordance with the segmentation scales and the principle of extraction from the easier to the advanced.

**Table 3:** Optimal segmentation scale parameter.

Object Level	Extract Information	Segmentation scale	Shape factor	Smooth
**Level1**	Forest	390	0.3	0.5
**Level2**	Water	130	0.3	0.5
**Level3**	Arable Land	190	0.5	0.5
**Level4**	Region	110	0.5	0.5

**Fig 4:**
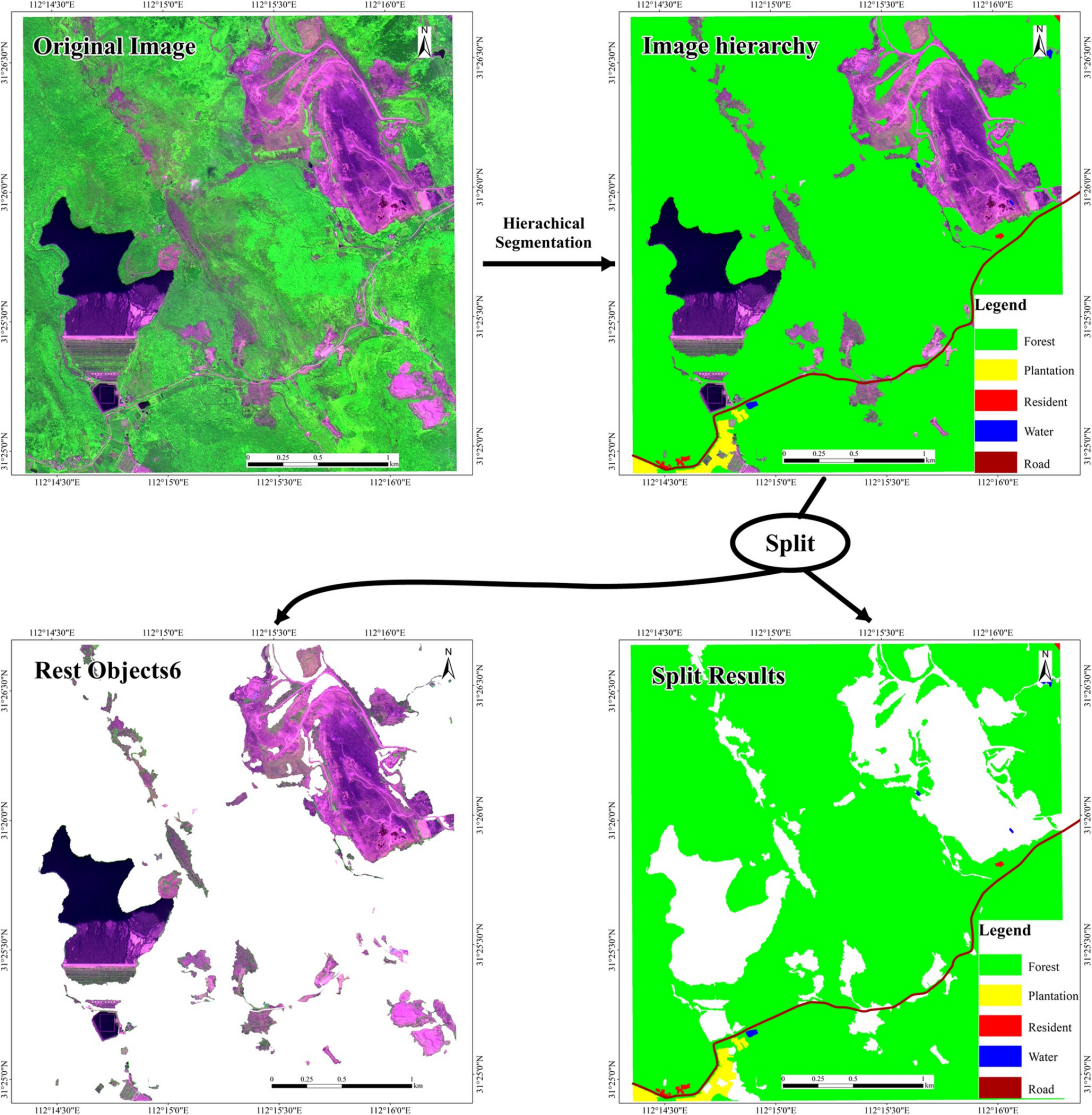
Differentiation layer extraction result graph of test area. Republished under a CC BY license, with permission from HBUT, original copyright 2017.

Then the information is extracted by using the fuzzy classification to set the membership function in eCognition based on the image characteristics of features and class-related characteristics among different features. Based on the classification hierarchy established above, the characteristics are selected to set the threshold of the membership function and establish corresponding fuzzy rules. The fuzzy rule library established in this paper is shown in Table 4. The results of the layered extraction are shown in Fig 4.

**Table 4:** Fuzzy rules.

Feature Type	Scale parameter	Classification threshold range
Forest	390, 0.3, 0.5	NDVI>0.42
Arable Land	190, 0.5, 0.5	GLCM StdDev>32
		Mean Nir>1750
		NDVI>0.26
		860<Mean Red<1470
Water	130, 0.3, 0.5	GLCM StdDev<30
		Men Nir<2270
		NDVI<0.13
Region	110, 0.5, 0.5	Brightness >800
		Compactness>500
		1.2<Density<2
		2.1<Shape Index<5
		0.8<Asymmetry<1
		0.5<Roundness<1.6
		110<Rectangular fit<180

Comment: NDVI—Normalized Differential Vegetation Index; GLCM StdDev—Standard Deviation of Gray Level Co-occurrence Matrix; Mean Nir—Mean value of Near Infrared band; Brightness—Brightness of each segmentation object; Compactness—Compactness of each segmentation object; Density—Densinty of each segmentation object; Shape Index—Shape Index of each segmentation object; Asymmetry—Asymmetry of each segmentation object; Roundness—Roundness of each segmentation object; Rectangular fit—Rectangular fit of each segmentation object.

### Extraction of mine development land information based on random forest model

In high-resolution remote sensing satellite images, different types of feature categories differ in their structure, material and texture. In addition, various features are mixed, interfering with the extraction of information on the features. Considering the complex diversity of feature object categories in high-resolution satellite images taken from remote sensing and the GF-2 image characteristics of the studied area, this paper selects a total of 23 characteristic factors from relevant spectral, geometric and texture characteristics to establish the characteristic factor sets for building the classifier model, as shown in Table 5.

**Table 5:** Feature sets.

Feature Type	Characteristic Parameters
**Spectrum**	Mean Gray value of each band (Total, Mean Layer 1, Mean Layer 2, Mean Layer 3, Mean Layer 4); Gray Standard Deviation of each band (Total, Standard deviation Layer 1, Standard deviation Layer 2, Standard deviation Layer 3, Standard deviation Layer 4); Normalized vegetation index (NDVI); Maximum difference (max diff)
**Shape**	Density; Shape index
**Texture**	Homogeneity of GLCM (Gray level co-occurrence matrix, GLCM); Contrast of GLCM; Entropy of GLCM; Mean of GLCM; StdDev of GLCM; Correlation of GLCM; Entropy of GLDV; Contrast of GLDV (Gray difference vector matrix, GLDV)

**Fig 5:**
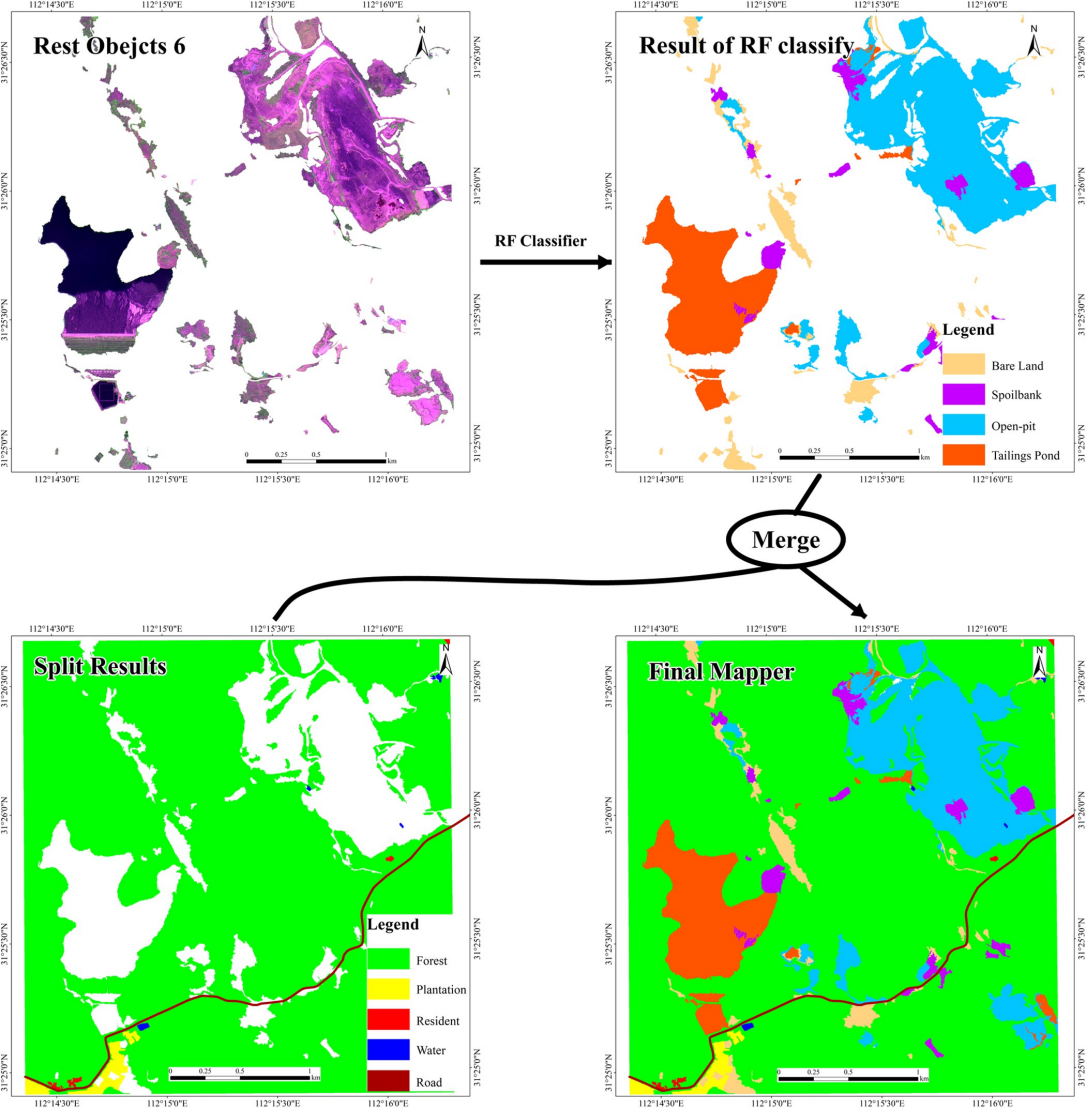
Extraction result of mine development land occupation information based on RF model. Republished under a CC BY license, with permission from HBUT, original copyright 2017.

As can be seen from the field survey data, main mining development land in the test area includes open-pit mines, tailing ponds, and waste slag piles. For the samples selected in the training area, the ED2 index is also used to obtain the optimal segmentation parameters to perform multi-scale segmentation. The segmentation results are merged into a vector layer. Each segmentation unit is assigned a value based on the GF-2 high-resolution fusion images and the field survey results for the open-pit mine development land. Finally, the training area data and the characteristic factor set are used to train the random forest model. The model is also used to extract different development land types of open-pit mines in the test area. Relevant information extraction results are shown in Fig 5.

## Results and analysis

### Importance analysis of characteristic factors

For the random forest model, the scores are calculated for the importance of characteristic factors by getting the Gini index through calculation in the process of building the model [33]. When the characteristic factor i is divided at the node, the current Gini index (DGi) will be calculated in the model. The Gini index minimization criterion is used to select the characteristics and determine the best binary split point of the characteristics. Finally, the average Gini index value of the characteristic factor in the model is calculated as the score index for the importance of the characteristic factor, as shown in Eq 1.

$$P_{k}=\frac{\sum_{i=1}^{n}\sum_{j=1}^{t}D_{G_{k_{ij}}}}{\sum_{k=1}^{m}\sum_{i=1}^{n}\sum_{j=1}^{t}D_{G_{k_{ij}}}}\times 100\%$$ (1)

where *m*, *n*, and *t* represent the number of characteristic factors, the number of decision trees, and the number of nodes in a single decision tree, respectively; $D_{G_{K_{ij}}}$ represents the Gini index value of the characteristic factor k at the *j*-th node of the *i*-th decision tree; *P*_*k*_ represents the importance score value of the characteristic factor k. The characteristic factor importance ranking of the RF model can be obtained through calculation, as shown in Table 6. The importance ranking of the characteristic factor of the RF model shows that NDVI is the most important factor among all characteristic factors, followed by the fourth band standard deviation, the mean Digital Number (DN) of the remote sensing image’s fourth band, the third band standard deviation and the third band DN mean of the GF-2 image.

**Table 6:** Feature factors importance ranking of RF model.

Characteristic factor	Weight	Characteristic factor	Weight
Normalized vegetation index (NDVI)	8.56%	Boundary index	2.42%
STD of the 4^**th**^ band DN value	8.12%	Mean of DN value	2.39%
Mean of the 4^**th**^ band DN value	7.50%	STD(Total)	2.37%
STD of the 3^**th**^ band DN value	6.13%	Roundness	2.36%
Mean of the 3^**th**^ band DN value	5.63%	Entropy of GLCM	2.32%
Mean of the 2^**nd**^ band DN value	5.22%	Rectangle fit	2.18%
STD of the 2^**nd**^ band DN value	4.60%	Similarity of GLCM	2.12%
STD of GLCM	4.25%	Homogeneity of GLCM	2.03%
Mean of the 1^**st**^ band DN value	3.55%	Asymmetry	1.72%
Density	3.20%	Ratio of length and width	1.02%
Lightness	3.15%	Contrast of GLDV	0.89%
Maximum difference value	3.10%	Entropy of GLDV	0.85%
STD of the 1^**st**^ band DN value	2.78%	Contrast of GLCM	0.83%
Shape	2.56%	Mean of GLDV	0.77%
Firmness	2.54%	Dissimilarity of GLCM	0.36%
Mean of GLCM	2.53%		

### Analysis of information extraction results

The real values and information extraction results obtained from field surveys are used to perform the accuracy evaluation of the waste slag piles (L1), the open-pit mines (L2), the tailing ponds (L3), and the bare land (L4). The accuracy table is shown in Table 7. The values filled in the columns in the accuracy table are the true value.

**Table 7:** Accuracy table of feature information extraction based on RF classifier.

	Observed	L1	L2	L3	L4	Total
Predicted						
**L1**		21	9	4	3	37
**L2**		3	175	0	8	186
**L3**		0	10	64	4	78
**L4**		3	4	0	49	56
**Overall accuracy**		0.86				
**Total Kappa**		0.78				

To analyze and compare the accuracy and scientificity of the extraction of land elements of open-pit mining production through the random forest model, the decision tree model is introduced in this paper for the comparation experiment. The same characteristic factor set is used to extract the mining land elements in the test area. The same evaluation method is also applied to evaluate the extraction results, as shown in Table 8.

**Table 8:** Accuracy table of feature information extraction based on CART classifier.

	Observed	L1	L2	L3	L4	Total
Predicted						
L1		19	17	5	7	48
L2		3	158	0	5	166
L3		1	15	63	9	88
L4		4	8	0	43	55
Overall accuracy		0.79				
Total Kappa		0.68				

By comparing the results shown in Tables 7 and 8, the following conclusions can be drawn:

1. The total accuracy and the Kappa coefficient of the information extraction results of the two models are 86% and 0.78, 79% and 0.68, respectively. The best extraction effect is obtained when the random forest model is used. The random forest model is designed such that collection is realized through the weak classifiers, the entire characteristic factor set is fully utilized, and the impact of the characteristic factor correlation on the model classification accuracy is reduced through bootstrap sampling, which can effectively ensure the classification accuracy.
2. Finally, with respect to land allocated for mining developments, the information extraction performance of the two models are as follows: (RF/CART, 88%/80%) for open-pit mines, (RF/CART, 78%/70%) for waste slag piles, and (RF/CART, 94%/93%) for tailing ponds. As can be seen from the information hierarchical structure, a fuzzy feature set containing the development land background elements in a large range is established, and the information is extracted after large-scale development land background elements such as woodland, residential land and cultivated land are removed using the layered mask method. This makes the image characteristics of the main development land elements more prominent and obvious. The extraction accuracy of the final information is good.
3. Through the analysis of the confusion matrix, it can be found that the waste slag piles are mainly misclassified into the segmentation units of bare land (RF/CART, 11%/15%/) and open-pit mines (RF/CART, 11%/11%). Open-pit mines are mainly misclassified as waste slag piles (RF/CART, 5%/9%) and tailing ponds (RF/CART, 5%/8%). There are many misclassifications in the classification results. During field surveying and verifications, open-pit mines cover some bare land and waste slag piles; in particular, open-pit mines are always accompanied by waste slag piles. Some features of bare land are quite similar to those of waste slag piles. As a result, there are errors in the final information extraction results of the model. However, the main categories of land earmarked for the development of open-pit mines are extracted to the maximum possible extent through the model, which is of great significance for the entire information extraction process for land earmarked for the development of open-pit mines.

## Conclusion

By studying the Jingxiang phosphorus mining area in Jingmen Hubei Province using domestic high-resolution GF-2 remote sensing imagery and IBM SPSS Statistics 21, ArcGIS 10.3.2, MATLAB R2014, ENVI 5.3 and eCognition Developer 9.2 software appplications, multi-scale segmentation is used in conjunction with tree models to study the interpretation of high-resolution remote sensing images taken for land earmarked for the development of open-pit mines. Main research outcomes and conclusions are as follows:

This paper applied an information extraction method based on high-resolution images obtained using remote sensing techniques to interpret remote images of open-pit mining development land. High-resolution remote sensing satellite images are segmented based on the hierarchical structure of image information using this method. The Euclidean Distance 2 Index method is applied to calculate the optimal segmentation parameters for different levels to extract the element information, while multi-scale segmentation and layered mask techniques are used to separate and extract main large-scale development land background elements and establish fuzzy rule sets. 23 characteristic factors are then selected from the spectral, geometric and texture characteristics to establish the characteristic factor sets for building the classifier model. Finally, the classification effects of the two machine learning methods in the studied area are compared and analyzed. A confusion matrix and the Kappa coefficient are used to evaluate the accuracy of the classification results. The experimental Kappa coefficient based on the random forest classifier is 86%, while the experimental Kappa coefficient based on the classification and regression tree classifier is 79%. This indicates that the method introduced in this paper can be applied to the preliminary extraction of information on open-pit mining land development, while also being conducive to locating and identifying the main open-pit mining land development elements.

By using the research method introduced in this paper, significant geological environment problems can be extracted in large places such as open-pit mines, metal mine tailing ponds, and industrial squares. In addition, the hierarchical structure of image information is used to prioritize the extraction of a wide range of geological environment elements, which is conducive to distinguishing industrial squares and residential land that easy to be confused, thus improving the accuracy of information extraction of industrial squares.

Because it is difficult to obtain relevant high-precision geological data, topographic data, and mineral data, using high-resolution remote sensing images to extract information related to geological environment elements from large open-pit mining production sites is of limited effect, as only the spectral, texture and shape characteristics of remote sensing images can be used. This is not sufficient to distinguish the geological environment elements (such as waste slag piles, bare land, and non-metal tailing ponds) that are of excessive similarities in image characteristics. To a certain extent, a combination of multi-scale segmentation technology, tree classification models and image information hierarchical models improves the accuracy of information extraction for major geological environment problem elements.
